# Irinotecan Plus S-1 Followed by Hepatectomy for a Patient with Initially Unresectable Colorectal Liver Metastases, Who Showed Severe Drug Rash with Oxaliplatin Plus 5-FU and Leucovorin (FOLFOX)

**DOI:** 10.1155/2014/906759

**Published:** 2014-06-17

**Authors:** Hiroyuki Komori, Toru Beppu, Yasuo Sakamoto, Yuji Miyamoto, Hiromitsu Hayashi, Katsunori Imai, Hidetoshi Nitta, Masayuki Watanabe, Hideo Baba

**Affiliations:** ^1^Department of Gastroenterological Surgery, Graduate School of Life Sciences, Kumamoto University, Honjyo 1-1-1, Chuo-ku, Kumamoto 860-8556, Japan; ^2^Department of Surgery, Kumamoto Shinto General Hospital, Shinyashiki 1-17-27, Chuo-ku, Kumamoto 962-8655, Japan; ^3^Department of Multidisciplinary Treatment for Gastroenterological Cancer, Kumamoto University Hospital, Honjyo 1-1-1, Chuo-ku, Kumamoto 860-8556, Japan

## Abstract

For unresectable colorectal liver metastases (CRLM), hepatic resection with or without chemotherapy is the only curative treatment that sufficiently achieves long-term survival. However, occasional severe allergic responses to anticancer drugs necessitate treatment discontinuation. A 45-year-old woman presented with metachronous unresectable colorectal liver metastases. Chemotherapy with oxaliplatin plus 5-FU and leucovorin (FOLFOX) was initiated, but severe allergic dermatitis developed after the second cycle. Although she reported no prior history of adverse reactions to tegafur-uracil, a drug lymphocyte stimulation test showed an allergic response to 5-FU. We subsequently replaced with Irinotecan plus S-1 (IRIS) chemotherapy which was well tolerated and resulted in a partial response after 3 cycles. As a result, right trisectionectomy was successfully performed and no recurrence was detected in the following 3 years. A severe allergic reaction to intravenous 5-FU-containing drug regimens can be successfully alleviated by switching to S-1-containing regimens such as IRIS or S-1 plus oxaliplatin (SOX).

## 1. Introduction

For unresectable colorectal liver metastases (CRLM), hepatic resection with or without chemotherapy is the only curative treatment that can yield 5-year survival rates of 20%–50% [1–4]. Therapeutic regimens including 5-FU plus irinotecan or oxaliplatin and biologic agents have recently been introduced [5]. 5-FU can be intravenously administered as a bolus- or as continuous infusion. Although it has been used as the main chemotherapeutic agent for colorectal cancer, it is associated with various adverse effects [6]. Allergic dermatitis can be caused by an immunological or nonimmunological response to drugs. The latter is usually dose related and can occur in otherwise normal individuals [7]. The difference in allergic responses between bolus and continuous 5-FU injections has not been fully elucidated. S-1 is an oral fluoropyrimidine preparation that combines tegafur with two 5-FU modulators 5-chloro-2,4-dihydropyrimidine (CDHP) and potassium oxonate, in 1 : 0.4 : 1 molar ratio. Although CDHP suppresses 5-FU degradation, thereby maintaining high 5-FU levels in plasma and tumor cells, peak plasma 5-FU levels are much lower than those achieved with bolus administration [8, 9]. In addition, drug-induced lymphocyte stimulation tests (DLST) are caused not only by anticancer drugs themselves but also by drug additives. Injectable 5-FU solution contains trometamol as a stabilizer, and this agent is not included in oral fluoropyrimidine preparations such as S-1 or tegafur-uracil UFT. Here we report the case of a 45-year-old woman with initially unresectable CRLM who developed an allergic reaction to oxaliplatin plus 5-FU and leucovorin (FOLFOX) and was switched to irinotecan plus S-1 (IRIS), prior to a successful conversion hepatectomy.

## 2. Case Presentation

A 45-year-old woman was referred to our hospital for the treatment of asymptomatic CRLM in February 2006. In May 2004, she underwent sigmoidectomy at an affiliated hospital and was diagnosed with colon cancer (well-differentiated adenocarcinoma). During followup, she developed dermatomyositis, necessitating steroid administration (prednisolone 5 mg every alternate day). In December 2005, she was diagnosed with unresectable CRLM because tumor free margins could not be achieved. She was administered a UFT regimen followed by FOLFOX4 because therapeutic effect of UFT was insufficient. After the second FOLFOX cycle, chemotherapy was ceased because of severe allergic dermatitis. DLST showed an allergic response to the 5-FU component of FOLFOX; she was referred to our hospital for additional treatment. Hematological tests performed at admission showed normal results. However, biochemistry tests revealed elevated asparate aminotransferase (44 U/L), alanine aminotransferase (63 U/L), lactate dehydrogenase (530 U/L), alkaline phosphatase (455 U/L), and gamma-guanosine triphosphate (192 U/L) levels. Carcinoembryonic antigen (CEA) levels were extremely high (596.9 ng/mL), indicating extensive CRLM.

**Figure 1:**
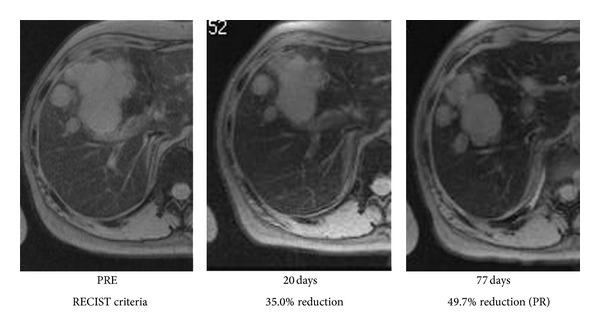
Evaluation of the size of liver metastases after chemotherapy by RECIST criteria. After 3 cycles of IRIS (77 days), the reduction in size of the tumor achieved 49.7%, which was interpreted as partial response.

**Figure 2:**
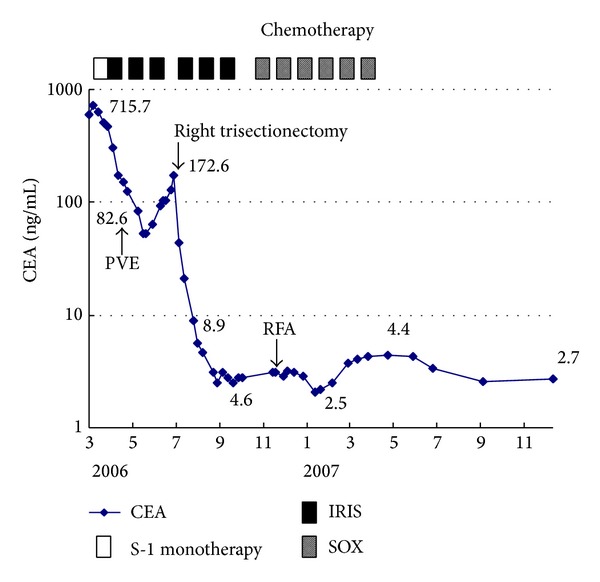
The time course of serum CEA level. S-1 monotherapy: S-1 120 mg/day for 2 weeks; IRIS: day 1 CPT-11 160 mg/body; TS-1 120 mg/body for 2 weeks followed by a 2 week rest period; SOX: day 1 Oxaliplatin 120 mg/body, TS-1 120 mg/body for 2 weeks followed by a 2-week-rest period. PVE: portal vein embolization; RFA: radiofrequency ablation therapy.

We thought that allergic dermatitis might be caused by high peak concentration of 5-FU resulting from 5-FU bolus or additives in the injectable 5-FU solution, because prior UFT administration had been successfully done without any adverse event. S-1 and UFT include the same effector of Tegafur which showed lower concentration of plasma 5-FU level. Additionally we could not deny the possibility of an allergic reaction to Oxaliplatin. After careful informed consent referring recurrence of allergic dermatitis, we changed regimen to IRIS therapy instead of modified FOLFOX therapy without bolus 5-FU administration. Initially, S-1 monotherapy (120 mg/day for 2 weeks) was administered to minimize adverse effects and assess its safety. After a 1-week drug-free interval, IRIS (CPT-11, 160 mg/body on day 1 and daily and TS-1, 120 mg/day for 2 weeks, followed by a 2-week-rest period) was initiated. After 3 IRIS cycles, partial response was observed (49.7%; RECIST criteria; Figure 1). CEA levels decreased from 596 to 123 ng/mL. Before the third IRIS cycle, portal vein embolization of the right portal branch was performed via a percutaneous ipsilateral approach using a gelatin sponge and ethanolamine oleate [10]. Liver regeneration of the unaffected lobe was sufficient, and there were no distant metastases or liver function impairment. After an additional IRIS cycle, right hepatic trisectionectomy was attempted. More than 5 tumors were found in the resected liver, the largest measuring 9.5 cm in diameter. Gallbladder invasion was also detected. A >1 cm surgical margin was achieved with no residual tumor detected on intraoperative ultrasound. No postoperative complications were observed; the patient was discharged on postoperation day 16. Histopathologically, the tumor was Grade 1b (1/3-2/3 viable), and there was no steatohepatitis in the noncancerous liver (NAFLD activity score, 2) [11]. Three additional IRIS cycles were administered (Figure 2). The symptoms of dermatomyositis did not get worsen during treatment. Five months after surgery, a small (1.6 cm) CRLM was detected in segment 3, necessitating percutaneous radiofrequency ablation and 6 S-1 plus oxaliplatin (SOX) cycles. Since then, she is being followed as an outpatient and has survived for more than 5 years from initial therapy for CRLM.

## 3. Discussion

5-FU has been the main chemotherapeutic agent against colorectal cancer for four decades, with modest efficacy [12]. Our patient developed severe 5-FU-induced allergic dermatitis, excluding the possibility of using this key drug. Although DLST clearly indicated an allergic response to 5-FU, the patient had tolerated UFT, a 5-FU prodrug, before FOLFOX therapy. We hypothesized two possibilities: high 5-FU levels due to bolus FOLFOX administration or the additives of injectable 5-FU solution. Peak plasma 5-FU levels range from 14,200 to 27,500 ng/mL after intravenous bolus injection (600 mg/m^2^) using modified de Gramont regimen [13]. S-1 is a well-known DIF drug that resulted in low peak 5-FU levels. Peak plasma 5-FU levels were reportedly 189–208 ng/mL following 50–60 mg/m^2^/day of S-1 administration [9]. Peak 5-FU levels after S-1 therapy were 100-fold lower than those after bolus 5-FU injection. Unfortunately, we did not measure plasma 5-FU levels in this case. Trometamol is an additive used to stabilize injectable 5-FU solutions and is not included in S-1 or UFT. It is used as a buffering agent, and few reports mention its association with allergic responses. Unfortunately, we did not conduct DLST for trometamol. To avoid the allergic dermatitis, we initiated IRIS therapy instead of the standard FOLFIRI regimen [14]. S-1 monotherapy for 2 weeks, followed by 3 IRIS cycles, was completed without serious adverse events.

IRIS therapy has shown a high response rate (50%–63%) in patients with metastatic colorectal cancer [15, 16]. In contrast, the IROX regimen (irinotecan-oxaliplatin combination without 5-FU) showed a relatively low response rate (22%) in a phase III study [17]: similarly, a SOX regimen showed a lower response rate of 31% to 69% [18]. The IRIS regimen in our patient resulted in partial response and was followed by successful curative resection for initially unresectable CRLM. Chemotherapeutic regimens containing CPT-11 have a significant association with steatohepatitis: these patients show a 10-fold increase in 90-day mortality compared with those without steatohepatitis [19]. 5-FU was also reportedly associated with steatosis or steatohepatitis in noncancerous tissues [20]. The resected liver specimen from our patient showed no steatohepatitis.

CRLM patients with severe allergy to 5-FU can be successfully treated with S-1-containing regimens such as IRIS or SOX. To avoid serious adverse effects, it is important to choose a suitable form of drug delivery and an effective chemotherapeutic regimen.
